# Olanzapine-Induced Thrombocytopenia in a Patient With Chronic Schizophrenia: A Case Report

**DOI:** 10.7759/cureus.74677

**Published:** 2024-11-28

**Authors:** Syed Ali Bokhari, Hanan Butaher, Reem Saleem, Nahid M.Elhassan

**Affiliations:** 1 Psychiatry, Al Amal Psychiatric Hospital, Emirates Health Services, Dubai, ARE

**Keywords:** antipsychotics, drug-induced thrombocytopenia, hematology, psychiatry, schizophrenia and other psychotic disorders, treatment-resistant schizophrenia

## Abstract

Olanzapine, a second-generation antipsychotic widely used for schizophrenia, is primarily known for its efficacy in managing both positive and negative symptoms. While its metabolic side effects are well-documented, hematologic complications such as thrombocytopenia are rare and often underrecognized. A 30-year-old Middle Eastern male with a longstanding history of schizophrenia developed persistent thrombocytopenia after several years of olanzapine use, with platelet counts consistently below the normal range. Despite being asymptomatic for bleeding or bruising, his platelet decline necessitated treatment adjustments. Cross-tapering olanzapine with other antipsychotics initially failed due to psychiatric relapse, but the introduction of aripiprazole alongside olanzapine tapering successfully improved platelet counts while maintaining psychiatric stability. The successful use of aripiprazole in this case represents a novel therapeutic approach, addressing antipsychotic-induced thrombocytopenia without compromising psychiatric outcomes. This case underscores the rare but significant risk of olanzapine-induced thrombocytopenia and highlights the need for vigilant hematologic monitoring during long-term antipsychotic therapy, even in asymptomatic patients or those with low baseline platelet count or concomitant blood dyscrasias.

## Introduction

Olanzapine, a second-generation atypical antipsychotic, is widely prescribed for managing schizophrenia and bipolar disorder due to its efficacy in addressing positive, negative, and mood symptoms. Its favourable safety profile compared to first-generation antipsychotics has led to extensive use in long-term therapy. However, olanzapine is associated with side effects, most notably metabolic disturbances such as weight gain and diabetes, which are well-documented. In contrast, its hematologic adverse effects, such as thrombocytopenia (a reduction in platelet count that increases bleeding risk) are rarely reported, with only 12 cases documented in the literature to date [1-12].

Thrombocytopenia is more commonly associated with antipsychotics such as clozapine, known for its risk of agranulocytosis and other blood dyscrasias. Although the exact mechanism of olanzapine-induced thrombocytopenia remains unclear, hypotheses include immune-mediated platelet destruction and direct bone marrow suppression, with some evidence pointing to structural similarities between olanzapine and clozapine as a possible contributing factor. This condition typically emerges after prolonged therapy and is often detected incidentally during routine blood tests, as patients frequently remain asymptomatic until significant platelet depletion occurs [2,7].

The reversibility of thrombocytopenia upon discontinuation of olanzapine underscores the importance of early detection through routine monitoring. This is clinically relevant for patients with pre-existing hematologic conditions or those on concurrent medications that may exacerbate hematologic toxicity. While current guidelines do not mandate routine blood counts for olanzapine, heightened vigilance may be warranted in at-risk populations. Management of olanzapine-induced thrombocytopenia often involves discontinuation of the offending agent, with corticosteroids or lithium employed in previous cases to restore platelet counts [1-3,5-7,8]. However, there remains a critical need for alternative therapeutic strategies that address thrombocytopenia without compromising psychiatric stability. This case report examines the use of aripiprazole alongside tapering olanzapine to a minimal effective dose as a novel approach to successfully increasing platelet counts, offering another potential solution for tackling this rare complication.

This case report highlights a unique instance of olanzapine-induced thrombocytopenia in a Middle Eastern male with chronic schizophrenia. By examining the clinical course, treatment adjustments, and outcomes, it emphasizes the importance of individualized care, innovative therapeutic strategies, and routine monitoring to balance psychiatric stability with the safe use of antipsychotic medications.

## Case presentation

A 30-year-old Middle Eastern male patient with chronic schizophrenia was initially admitted to a general hospital with psychiatric services at the age of 20. He presented with predominantly negative symptoms, including diminished initiative, self-neglect, poverty of speech, and occasional episodes of inappropriate laughter. Following his initial hospitalization, he was transferred to our tertiary psychiatric facility, where he has received ongoing care. Diagnosed during his high school years, his illness has been marked by frequent relapses and remissions. There was no reported family history of mental illness or medical illness, including haematological disorders.

Upon his initial diagnosis in 2011, the patient was prescribed olanzapine 20 mg daily, sodium valproate 500 mg once daily, and long-acting risperidone injections at 50 mg twice monthly. Despite this regimen, he continued to experience auditory hallucinations, with his family observing aggressive behaviour and social withdrawal. Negative symptoms, such as withdrawal from family interactions and lack of motivation, became more pronounced, accompanied by reports of hearing voices and hallucinatory behaviour.

In 2014, one year after admission, the patient experienced his first seizure, which was managed with ongoing sodium valproate therapy. Despite persistent psychotic and negative symptoms, his condition remained relatively stable, with periodic fluctuations.

**Figure 1:**
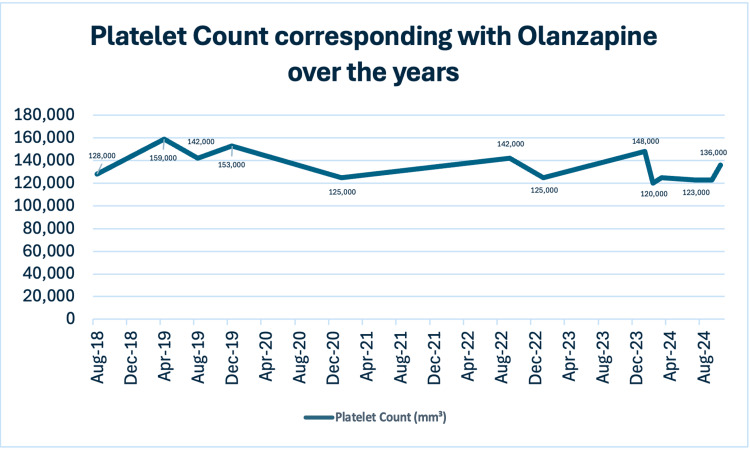
Platelet counts over the years.

By August 2018, routine blood tests revealed a drop in platelet count to 128,000/mm³, slightly below the normal range. This marked the beginning of thrombocytopenia, although the patient remained asymptomatic, with no signs of bleeding or bruising (Figure 1). To investigate the cause of thrombocytopenia, complete blood count (CBC) revealed reduced platelet levels without changes in haemoglobin or white blood cell counts. A peripheral blood smear showed no abnormal cell morphology, excluding intravascular hemolysis. Coagulation studies, including prothrombin time (PT) and activated partial thromboplastin time (aPTT), were within normal limits. Liver function tests showed no abnormalities, reducing the likelihood of significant liver disease or portal hypertension. Urine analysis showed no evidence of hematuria or proteinuria. Although an abdominal ultrasound was not performed, clinical findings did not indicate splenomegaly or other signs of portal hypertension.

In August 2019, due to concerns over olanzapine’s metabolic side effects (weight gain and suspected fatty liver), olanzapine was tapered to 5 mg daily and eventually discontinued. Aripiprazole was introduced at 10 mg daily and then increased to 20 mg daily after a week. During this period, the patient’s platelet count rose to 153,000/mm³, reflecting improvement after discontinuing olanzapine. However, by September 2019, a psychiatric relapse occurred, with increased auditory hallucinations and irritability. Consequently, aripiprazole was discontinued, and olanzapine was reintroduced at 10 mg daily. Shortly after, his platelet count dropped to 142,000/mm³, supporting the likelihood of olanzapine-induced thrombocytopenia.

In January 2021, the patient developed obsessive-compulsive behaviours, including excessive handwashing. Fluoxetine 20 mg daily was added to address these symptoms. Although his platelet count remained low at 125,000/mm³, he showed no physical signs of thrombocytopenia such as purpura, petechiae, prolonged bleeding from cuts, gum bleeding, nosebleeds, hematuria, melena, and fatigue. His psychiatric symptoms were stable, and his olanzapine dose was maintained at 20 mg daily, alongside long-acting risperidone injections.

Throughout 2022, the patient continued on olanzapine 20 mg daily, paliperidone 150 mg intramuscular (IM) monthly, and sodium valproate 500 mg daily. His platelet count in September 2022 was 142,000/mm³, still within the low range. He remained clinically stable, although persistent negative symptoms, such as lack of motivation and poverty of speech, were noted. His mother reported reduced engagement with the family despite improvements in obsessive-compulsive symptoms with fluoxetine, which was increased to 40 mg daily.

By January 2023, his platelet count dropped again to 125,000/mm³, though he remained asymptomatic. His psychiatric symptoms, including obsessive compulsive disorder, although persistent, were stable.

In January 2024, his platelet count continued to fluctuate, ranging from 153,000/mm³ in January to 120,000/mm³ in February, with a reading of 125,000/mm³ in March. Concerns over olanzapine’s metabolic side effects prompted a reassessment. In May 2024, olanzapine was reduced to 10 mg daily, and aripiprazole was introduced at 5 mg daily. Long-acting Paliperidone IM injections at 150 mg monthly were continued. Despite persistent thrombocytopenia, the patient denied bleeding or bruising and showed improvement in his negative symptoms.

By September 2024, his platelet count had dropped to 123,000/mm³. Aripiprazole was increased to 15 mg daily to address thrombocytopenia. A follow-up complete blood count (CBC) in October 2024 showed an improvement in platelet count to 136,000/mm³, marking a 10.5% increase. This hematologic recovery further supported the likelihood of olanzapine-induced thrombocytopenia, correlating with the reduced olanzapine dose. 

The patient remains under close monitoring for both psychiatric stability and thrombocytopenia. His current regimen, in November 2024, consists of olanzapine 5 mg daily, aripiprazole 20 mg daily, fluoxetine 40 mg daily, and long-acting paliperidone 150 mg intramuscular (IM) monthly. Although thrombocytopenia persists, no clinical signs of bleeding or bruising have been observed, and his obsessive-compulsive symptoms remain well-controlled. This improvement demonstrates the efficacy of dose adjustments and cross-tapering strategies in managing olanzapine-induced thrombocytopenia while maintaining psychiatric stability.

## Discussion

Olanzapine-induced thrombocytopenia is a rare but significant adverse effect, with only 12 cases reported in the literature [1-12]. While thrombocytopenia is more commonly linked to clozapine, the documentation of similar haematologic complications with olanzapine raises concerns about its long-term safety. The proposed mechanisms include immune-mediated platelet destruction, where olanzapine forms a neoantigen-triggering platelet depletion and direct suppression of bone marrow function. The structural similarity between clozapine and olanzapine suggests that patients sensitive to one may be at risk of the other [2,7]. 

The current case contributes to this growing body of evidence by demonstrating recurring thrombocytopenia after years of olanzapine use. While the thrombocytopenia observed in this case was mild and asymptomatic, its persistence over several years and consistent correlation with olanzapine use highlight its clinical relevance. Persistent thrombocytopenia, even when asymptomatic, signals an ongoing hematologic effect that requires close monitoring to prevent potential progression or complications, especially in patients on long-term antipsychotic therapy. 

Platelet counts fluctuated between 120,000 and 153,000/mm³, with improvement observed upon cross-tapering olanzapine to the minimal effective dose with aripiprazole, which was gradually increased to 20 mg once daily. Unlike previous reports where interventions such as corticosteroids or lithium were employed to address antipsychotic-induced hematologic complications, this case is unique in demonstrating that aripiprazole, introduced alongside a reduction in olanzapine dosage, successfully increased platelet counts without necessitating the discontinuation of antipsychotic therapy. This finding highlights aripiprazole as a potential novel therapeutic alternative for managing olanzapine-induced thrombocytopenia, particularly when maintaining psychiatric stability is paramount.

Prior cases have reported the utility of lithium in stabilizing blood counts, particularly in individuals who developed thrombocytopenia with both clozapine and olanzapine [7,8]. Similarly, corticosteroids have been used to counter immune-mediated platelet destruction, albeit with variable success. However, the approach in this case - gradually tapering olanzapine to the lowest effective dose while introducing aripiprazole - offers an innovative solution by simultaneously addressing both hematologic and psychiatric needs. This strategy provides a practical alternative for managing thrombocytopenia in cases where immediate cessation of olanzapine is either impractical or risks exacerbating psychiatric symptoms [3,6].

Notably, the patient in this case remained asymptomatic for bleeding or bruising despite significant platelet fluctuations, underscoring the silent nature of this adverse effect and the need for routine hematologic monitoring in patients undergoing long-term olanzapine therapy. Current guidelines do not mandate such monitoring; however, periodic blood counts should be considered for high-risk patients, such as those with concurrent medications that affect platelet function or a history of hematologic conditions [7,8]. Educating patients and families about early warning signs, such as unexplained bruising or bleeding, remains critical for timely detection and intervention [6,7].

Given the rarity of this condition, further research is essential to elucidate its mechanisms and identify patient-specific risk factors. Longitudinal studies with representative samples could provide valuable insights, guiding clinicians on when and how to implement routine monitoring. Until such data become available, a cautious approach that prioritizes hematologic monitoring in at-risk populations is recommended. This case underscores the importance of individualized care, illustrating how innovative therapeutic strategies can effectively balance hematologic safety and psychiatric stability [1,7].

This case highlights the successful use of aripiprazole alongside olanzapine dose tapering as an effective intervention for olanzapine-induced thrombocytopenia. This approach offers another potential solution for managing this rare adverse effect, contributing to the growing repertoire of strategies for tackling antipsychotic-induced hematologic complications. Clinicians must remain vigilant and adapt treatment plans to individual needs, ensuring safe and effective care for patients with chronic psychiatric conditions.

## Conclusions

This case highlights the critical need for vigilant monitoring in patients undergoing long-term olanzapine therapy, as it demonstrates the potential for olanzapine-induced thrombocytopenia to persist silently without overt clinical symptoms, such as bleeding. The gradual decline in platelet counts underscores the risk of this adverse effect being missed without routine blood work. Early detection through regular monitoring enables clinicians to adapt treatment regimens, balancing the need to maintain psychiatric stability while mitigating haematologic risks. Regular follow-up, ongoing evaluation, and close collaboration with the patient and family are essential to ensuring both psychiatric stability and overall health, as well as the early identification of potential side effects.

## References

[1] Bogunovic O, Viswanathan R (2000). Thrombocytopenia possibly associated with olanzapine and subsequently with benztropine mesylate. Psychosomatics.

[2] Bachmann S, Schröder J, Pantel J, Mundt C, Zorn M, Witzens M, Egerer G (1998). Olanzapine-induced thrombocytopenia in association with idiopathic thrombocytopenic purpura. Br J Psychiatry.

[3] Onofrj M, Thomas A (2001). One further case of pancytopenia induced by olanzapine in a Parkinson's disease patient. Eur Neurol.

[4] Tu CH, Yang S (2002). Olanzapine-induced EDTA-dependent pseudothrombocytopenia. Psychosomatics.

[5] Carrillo JA, González JA, Gervasini G, López R, Fernández MA, Núñez GM (2004). Thrombocytopenia and fatality associated with olanzapine. Eur J Clin Pharmacol.

[6] Rai S, Chakrabarti S, Lobana A (2004). Pancytopenia on switching from clozapine to olanzapine: a case report and some unresolved issues. Indian J Pharmacol.

[7] Mehta A, Sanitato J (2005). A case of neutropenia and thrombocytopenia shortly after initiating olanzapine. Psychiatry (Edgmont).

[8] Grover S, Hegde A, Agarwal M, Sachdeva MS (2012). Olanzapine-associated leukopenia and thrombocytopenia managed with lithium in a patient who developed leukopenia with clozapine in the past: a case report. Prim Care Companion CNS Disord.

[9] Maurier F, Petitpain N, Guichard JF, Javot L, Tréchot P (2010). Olanzapine and pancytopenia with severe folate deficiency. Eur J Clin Pharmacol.

[10] Sahoo S, Singla H, Spoorty M, Malhotra P, Grover S (2016). Thrombocytopenia associated with olanzapine: A case report and review of literature. Indian J Psychiatry.

[11] Lin YH, Chien YL (2012). Valproic Acid-related anticonvulsant hypersensitivity syndrome and subsequent olanzapine-related neutropenia and thrombocytopenia: a case report. J Clin Psychopharmacol.

[12] Cruz MD, Danoff R (2017). Thrombocytopenia and spontaneous intracranial hemorrhage after olanzapine therapy. J Am Osteopath Assoc.

